# Health physics workforce in the United States

**DOI:** 10.1002/acm2.13757

**Published:** 2023-01-27

**Authors:** Michael A. Noska, Caridad Borrás, E. Vincent Holahan, Shaheen A. Dewji, Thomas E. Johnson, Jerry W. Hiatt, Wayne D. Newhauser, John W. Poston, Nolan Hertel

**Affiliations:** ^1^ U.S. Food & Drug Administration (retired) Falls Church Virginia USA; ^2^ Radiological Physics and Health Services Georgetown University Washington District of Columbia USA; ^3^ U.S. Nuclear Regulatory Commission Washington District of Columbia USA; ^4^ Nuclear and Radiological Engineering and Medical Physics Programs, George W. Woodruff School of Mechanical Engineering Georgia Institute of Technology Atlanta Georgia USA; ^5^ Environmental and Radiological Health Sciences Colorado State University Fort Collins Colorado USA; ^6^ Nuclear Energy Institute Washington District of Columbia USA; ^7^ Department of Physics & Astronomy Louisiana State University Baton Rouge Louisiana USA; ^8^ Department of Physics & Astronomy Mary Bird Perkins Cancer Center Baton Rouge Louisiana USA; ^9^ Texas A&M University College Station Texas USA; ^10^ Nuclear and Radiological Engineering and Medical Physics Georgia Institute of Technology Atlanta Georgia USA

**Keywords:** health physics, workforce

## 2.1 INTRODUCTION

Health physics is an applied science field focused on the protection of workers, patients, the general public, and the environment from ionizing and nonionizing radiation sources. The role of the health physicist (HP), and the recognition of health physics as a distinct field, may be traced to the advent of nuclear power and its application after the Manhattan Project in the 1940s. The field grew steadily in size to meet the growing uses of nuclear technology until reaching a peak in the mid‐1990s.^1^ Subsequent shrinkage has been attributed mainly to attrition due to retiring baby boomers, a chronic decline in the number of replacement workers emerging from health physics education programs, and a reduced demand for health physics work in the nuclear power industry. Workforce shortages have been predicted,^2^, ^3^ although not confirmed in the literature.

## 2.2 DEFINITIONS OF THE PROFESSION

The discipline of health physics is devoted to the protection of people and their environment from the harmful effects of ionizing and nonionizing radiation while enabling its beneficial uses. Broadly, health physics may be considered an allied health profession with a diverse scope of practice, including essential activities in medicine, research, industry, education, emergency preparedness, radioactive waste management, environmental protection, regulation, and many other niche fields, such as radiation protection for national defense, civil aviation, and spaceflight. The International Labour Organization has recognized health physics professionals as radiation protection experts,^4^ and several radiation protection organizations, such as the Health Physics Society (HPS)^5^ and the International Radiation Protection Association, have articulated the basic aspects of the profession.^6^

For the purposes of this review, we define three categories of HPs, stratified by the level of attained qualifications:

- An “HP” has earned a BS or higher degree, has some work experience, but does not hold professional certification or a license to practice the profession. Suitable degrees include health physics, medical physics, physics, nuclear engineering, radiological science, or another closely related physical science;
- An “eligible health physicist” is any HP who meets established requirements for eligibility for professional certification or licensure;
- A “board‐certified health physicist ” is any HP who has fully met all of the applicable requirements for professional board certification.

In describing HPs, it is convenient to categorize them by the nature of their work (e.g., healthcare and regulatory compliance) and the type of employer (e.g., private sector and government). In terms of types of jobs and employers, the health physics workforce is broadly distributed. Although an HP is usually specialized in one area, many HPs perform duties across several areas and are able to move between employment sectors. Some of the most important functions performed by HPs are described as follows:

- Many HPs ensure the safe and secure use of nuclear materials in the nuclear fuel cycle, including the mining and milling of uranium; uranium enrichment; reactor fuel fabrication; electric power generation; the transport, storage, and disposal of used fuel; and the final decontamination and decommissioning of nuclear fuel cycle facilities. HPs, radiation protection managers (RPMs), and health physics technicians are employed at all stages of the nuclear fuel cycle to ensure worker and public health and safety, the safety and security of the nuclear material, and protection of the environment.
- Environmental HPs generally specialize in the evaluation of effluents from fuel cycle facilities and other facilities that utilize radioactive materials (e.g., pharmaceutical manufacturing), remediation and decommissioning of radioactive material facilities, mining and mineral extraction facilities, as well as the environmental impact of the use of radioactive materials. They model the movement of radioactive materials in the environment, the concentration of radionuclides at each trophic level, and evaluate the overall effect of remediation on the environment. Evaluation of the biological impact of exposure to radioactive materials on humans, fauna, and flora is an essential part of the environmental HP's work.
- The medical HP plays a key role in the safety of workers, patients, and the public from medical uses of radioactive materials and machine‐produced radiation. Medical HPs may also serve as radiation safety officers (RSOs) in hospitals. The RSO is responsible for overseeing and ensuring the safe operation of a hospital's radiation protection program, including managing the radiation protection program; identifying radiation protection problems and stopping unsafe activities; initiating, recommending, or providing corrective actions; verifying implementation of corrective actions; and ensuring compliance with federal and state regulations for safety and security. The training and experience requirements for a hospital RSO are described in Title 10 (Energy) of the Code of Federal Regulations (CFR 35.50). The RSO also may be required to evaluate and make recommendations regarding the potential hazards associated with nonionizing radiation sources (e.g., microwaves and lasers).
- A university or industrial HP is concerned with the management and control of radiation sources used in academic research, manufacturing, and other industrial applications, including radioactive materials and machine‐produced radiation sources. They operate under the regulatory authority of the Nuclear Regulatory Commission, Environment Protection Agency, Department of Energy (DOE), Occupational Safety and Health Administration, and/or a state radiation control program. Their duties include monitoring operations to ensure regulatory compliance and the safety of workers, the general public, and the environment. Academic HPs may teach undergraduate and graduate courses, perform research, and engage in other scholarly activities.
- Military HPs perform a wide variety of duties that encompass the diverse missions of the military's radiation safety programs, including those pertaining to weapons, propulsion, basic and applied research, radiation medicine, and emergency response. The military utilizes virtually all types of radiation sources, both ionizing and nonionizing, and the military HP engages in many of the programs earlier over the course of their careers.
- A large number of HPs are employed by state and federal regulatory agencies. They are engaged in establishing and enforcing the rules and regulations for the safe and secure manufacture, use and disposal of radioactive materials, and the uses of radiation‐producing devices in industry and medicine. They also are engaged in the issuance of licenses and permits to receive, possess, use, transfer, own or acquire radioactive material, and in the inspection of regulated facilities to ensure compliance with regulations and safety requirements.

## 2.3 GENERAL CHARACTERISTICS OF THE WORKFORCE

The exact size of the domestic professional health physics workforce has been estimated at 3200 and 7000 persons, depending on the definition used for HP and other factors. It is common for professionals from other radiation and nuclear disciplines, as well as industrial hygienists, to hold health physics positions. In 2004, the HPS estimated that ∼6700 radiation protection professionals are involved in radiation safety activities in the United States, with as many as 50% of these individuals being in activities associated with nuclear energy production.^7^ A later article from the Bureau of Labor Statistics in 2011 referenced an HPS estimate of the health physics workforce as being “more than 6500.” ^8^ The Oak Ridge Institute for Science and Education estimated that 4800 HPs were employed in the United States in 2009 (excluding medical facilities and other industries). “Based on very rough estimates,” another 2000 HPs may be employed in medical and health‐care facilities and other industries.^9^

Limited data are available on the variation of professional qualifications of radiation protection staff employed in the United States. For example, a survey by the Nuclear Energy Institute of all nuclear electric‐generating stations in the United States revealed that only 10% of 3800 radiation protection staff (including permanent and temporary, as well as professional and other types) required a 4‐year college degree in health physics or a related field. Only the position of RPM (HP) at each facility required a 4‐year degree (there are methods available to obtain the equivalent of a degree to satisfy this requirement) and a minimum of 5 years of experience, including 3 years of nuclear power plant experience directly related to the radiation protection program.^10^ The DOE's National Laboratories and its contractors have a total of ∼400 full time professional HPs employed at their facilities; these positions generally require a 4‐year degree in health physics or a related discipline. These data of professional requirements should be interpreted with caution because they are based on limited data. Neither the U.S. Department of Labor nor the Census Bureau collects employment data specifically for HPs but includes them in the broader category of physicists.

Data are available on the distribution of the health physics workforce by worker age, with over 50% of the HPS members being over 50 years of age, and with many planning to retire within 10 years.^1^, ^11^ Thus, many workers are close to, or already eligible for, retirement. The exact numbers of practicing HPs is not available, but the membership size of HPS (3100 in 2020) and the number of holders of board certification (1300) provide surrogate data. The HPS membership has decreased by 14% since 2003; this has been mainly attributed to a wave of retiring baby boomers and a comparatively small number of replacements entering the profession. Interestingly, over the same time period, the number of board‐certified health physicists has remained constant, that is, with ∼1300 holders of certificates from the American Board of Health Physics (ABHP). The decline in membership in the HPS is more severe than in societies of closely related professions; for example, the membership of the American Nuclear Society shrank by only 5% over the past 15 years.^1^ The current declared membership of HPS of ∼3100 is likely an underestimate, as not all HPs are members of the HPS.^12^

## 2.4 EDUCATION AND TRAINING PATHWAYS

Health physics is an applied branch of physics that is highly multidisciplinary (see Section 2.2). HPs enter the profession through traditional degree programs in health physics or a variety of alternate pathways. The educational entry pathways vary considerably with subspecialty, employer, and job responsibilities. As defined in Section 2.2, all types of professional HPs require at least a BS degree or higher in health physics or related science, with some positions requiring an MS or PhD degree.

### 2.4.1 Organizations involved in education

The Accreditation Board for Engineering and Technology (ABET) provides standards for higher education. The ABET health physics standards for education outcomes and objectives were developed by the HPS. There is considerable overlap between health physics and medical physics, especially in the subspecialty of medical health physics. The HPS lists health physics–related academic programs, including 31 schools offering a range of degrees from the BS to the PhD degrees (https://hps.org).

### 2.4.2 Undergraduate education

A professional HP holds a bachelor's (BS) or higher degree in physics, health physics, nuclear engineering, or in another biological or physical science. There are ∼18 regionally accredited colleges or universities in the United States that offer bachelor degree programs in health physics,^13^, ^14^ three of which are accredited by the ABET (Idaho State University, Oregon State University, and the University of Massachusetts at Lowell).^15^ Undergraduate programs in health physics are generally located within physics and nuclear engineering departments. Some programs offer an optional undergraduate concentration in health physics or nuclear science. Undergraduate curricula focus on breadth of knowledge and, typically, students interested in health physics will take only a few courses specifically related to health physics.

### 2.4.3 Graduate education

Graduate education available in health physics includes MS and PhD degree programs, as well as related degrees programs in biological or physical sciences and engineering. All graduate degrees in health physics count toward the requirements of an HP (see Section 2.4). Approximately 23 programs grant MS degrees and 17 grant PhDs.^14^ The curricula for MS degree programs vary substantially across the United States, although minimum standards for accreditation of an MS and PhD degree programs in health physics have been established by ABET (www.abet.org). These include classes in radiation physics, radiation biology, radiation detection and measurements with laboratory experience, internal and external radiation dosimetry, principles of radiation safety and health physics, and contemporary issues in health physics. As of 2020, only six graduate programs in health physics in the United States were ABET‐accredited (Clemson University, Colorado State University, Idaho State University, University of Massachusetts at Lowell, Oregon State University, and the University of Nevada at Las Vegas). In 2016, ∼20 accredited colleges and universities granted 66 master's degrees and 23 doctoral degrees in health physics or radiological sciences.^16^ Table 1 lists health physics graduates by degree and post‐graduation status, showing relative stability in the number of master's degrees conferred, but a drop in the number of doctoral degrees to 13.^17^ Currently, accreditation is not available for doctoral health physics curricula.

**TABLE 1:** Number of BS, MS, and PhD degrees in health physics conferred in the United States between September 2019 and August 2020^17^

Curriculum	BS	MS	PhD
Continued study/post‐doc	13	6	0
Academic employment	1	5	2
Federal government employment	0	5	0
DOE contractor employment	1	5	1
State and local government employment	0	1	0
Medical facilities employment	1	8	2
Nuclear utility employment	1	0	0
Other nuclear‐related employment	7	18	1
Other business employment	2	0	0
Foreign (non‐U.S.) employment	0	0	4
U.S. military, active duty	0	1	3
Unknown/not reported	14	18	0
**Total**	**40**	**67**	**13**

### 2.4.4 Postgraduate training

Formal postgraduate training programs in health physics are scarce. A limited number of postgraduate training opportunities in health physics are available from US national laboratories. It is worth noting that this situation comprises a radical change with past practices; postgraduate training was used extensively from the 1950s to the 1970s, especially by the U.S. Public Health Service.

### 2.4.5 Alternate pathways

Consensus requirements for alternative pathways into the profession of health physics are lacking, and the subject is controversial. Multiple alternative pathways exist, including the promotion of radiation control technicians, the U.S. Navy Nuclear Propulsion program, the Army public health training program, USAF Bioenvironmental Engineer training programs, DOE national laboratory training programs, and civilian training programs at nuclear power plants and shipyards. Alternative training in radiation protection may be obtained through courses offered by Oak Ridge Associated Universities (ORAU) and a number of commercial vendors, as well as courses sponsored by other federal agencies. Since 1948, ORAU has trained more than 30 000 scientists, physicians, engineers, educators, regulators, and personnel in a variety of radiation safety and health physics topics through its Professional Training Programs. It currently offers 16 courses at its training facility at Oak Ridge, TN. The NRC also offers 27 online and classroom radiation safety and health physics courses for staff employed by the NRC and NRC Agreement States. Course topics range from fundamental and advanced health physics to diagnostic and therapeutic nuclear medicine. These types of courses augment a 4‐year degree as an alternate pathway into the field of health physics. Basic RSO courses are commercially available that meet federal and state regulations and range from 8 to 40 h each.

Associate degree programs typically prepare graduates for careers as health physics technicians. Through the industry‐initiated Nuclear Uniform Curriculum Program, the commercial nuclear power industry has developed partnerships with multiple 2‐year degree programs, including one in radiation safety.^18^

### 2.4.6 On‐the‐job training

In addition to a BS or higher degree, an HP must obtain applied radiation protection experience by working under the supervision of an experienced HP, although the type and amount of experience needed varies considerably. For example, the requirements for an RSO at a panoramic sterilization irradiation facility include just 3 months of supervised occupational experience, whereas the requirements for a hospital RSO include 5 years of professional experience in health physics. At a U.S. nuclear power plant, no prior work experience or on‐the‐job training is required, whereas an RPM in the same facility requires a minimum of 5 years of related experience, which must include 3 years of nuclear power plant experience and 1 year of supervisory or management experience.^10^

### 2.4.7 Certification and licensure

Some positions in health physics require additional professional board certification and/or licensure. The ABHP is the main certification body for the practice of professional health physics in the United States, and it is responsible for establishing the qualifications for designation as a certified health physicist (CHP), as well as examining applicants. Requirements to become a CHP include education, work experience, and successful completion of a two‐part certification examination. HPs with a graduate degree can also apply to become a “Certified Medical Health Physicist” by the American Board of Medical Physics (ABMP). The ABMP^19^ offers certification in Medical Health Physics for diplomates that have, by examination, been issued certificates with the words “RSO Eligible” on their certificate. Similarly, the American Board of Radiology currently provides “RSO Eligible” status to diplomates in the Diagnostic Medical Physics and the Nuclear Medical Physics subspecialties (this certification will cease in 2023). The American Board of Science in Nuclear Medicine offers certificates in the Radiation Protection Specialty.

### 2.4.8 Continuing education

CHPs are required to obtain 80 h of continuing education every 4 years. CHPs have many options to obtain continuing education, as detailed on the American Academy of Health Physics (AAHP) website.^20^ The HPS, AAHP, and other organizations provide continuing education courses.

## 2.5 CURRENT STATUS AND FUTURE OUTLOOK

The current status of the health physics workforce is difficult to discern because of insufficient data on the supply and demand for health physics services. Despite apparent declines in the number of HPs, the current workforce appears to meet the needs for radiation protection across all sectors, mainly because eligible workers have delayed their retirements due to the uneven performance of the economy since 2008. However, it is generally accepted that the number of HPs will decline significantly in the near future due to the retirements of baby boomers.

Predictions for future sustainability of the workforce are less certain. The NCRP concluded in its Statement 12^3^ that the nation will face a severe shortage of radiation professionals that could jeopardize national security without mitigation. A 2017 HPS publication pointed out that the number of health physics graduates had declined by 55% from 1995 to 2015 and predicted that future supply will not meet demand.^20^ On the other hand, one author suggested that the health physics profession may be a victim of its own success in that radiation protection programs have become so effective as to be capable of functioning with safety generalists replacing (higher cost) HPs.^21^ Further, this author referenced the public's aversion to radiation and nuclear technology, along with the closure of numerous civilian nuclear power plants, as reasons to predict a stagnant or diminished demand for radiation protection specialists in the future. He did cite three potential areas for growth—decommissioning, environmental protection, and medicine—and recommended a strengthening of standards for health physics education, training and experience, and improved outreach to attract students to the field.

The field of radiological emergency preparedness has taken on increased importance since the events of 9/11, as the threat of intentional destruction and widespread contamination with radiological or nuclear devices has increased. The nuclear disaster in Fukushima, along with the earlier accidents at Chernobyl and Three Mile Island, illustrate the need to maintain a viable cadre of highly trained radiation specialists to respond to the effects of such radiologic accidents. However, the low probability of these types of incidents creates a conundrum for employers and government leaders, as it is generally not cost‐effective to maintain staff for the sole purpose of responding to such emergencies. Therefore, emergency preparedness and response are typically collateral duties for HPs. However, the lack of surge capacity for large‐scale incidents represents a significant gap in the safety and security of the nation and highlights the need for specialized training.

With respect to the civilian nuclear power industry, power plants require a total of ∼3700 radiation protection specialists, 400 of whom would be classified as HPs.^7^ A recent study revealed that current needs are being met for full‐time nuclear power utility staffing, that is, professional HPs and technicians. This finding was based on human resource data from a 2015 survey, using projected retirement and attrition data and the projected supply from 2‐year institutions and 4‐year advanced degree programs.^22^ The apparent adequacy of the workforce differed from earlier predictions of possible shortages.^2^, ^3^ A possible explanation is that the long‐anticipated renaissance of the nuclear industry did not occur; only two new nuclear power reactors are under construction in the United States in 2021. In addition, since 2013, 13 nuclear power plants have permanently ceased operations, and several utilities have announced that additional units, including Byron 1 and 2, Dresden 1 and 2, Palisades, and Diablo Canyon 1 and 2, will close by 2025. As plant construction, closures, and decommissioning significantly impact the demand for HPs, the future needs in the nuclear power industry are difficult to forecast with certainty. The US Bureau of Labor Statistics predicts that employment in the nuclear sector will decline by ∼20% over the current decade; however, some of these losses may be offset by an increased demand for decontamination and decommissioning services.^23^

The status of the health physics workforce employed in the medical sector is unclear. It is difficult to estimate the number of HPs with specialization in medical health physics because of overlapping job responsibilities with medical physicists (see Chapter 3). Demand for medical health physics is difficult to forecast because it is driven by regulatory requirements, the size and age of the population, the utilization of radiation in medicine, productivity, and health‐care economics. However, due to technological innovations and the medical needs of an aging population, it can be expected that this sector would experience stable or slightly increased employment.

The federal government, the largest single employer of HPs, decreased its ranks from 451 HPs in 2004 to 418 HPs in 2016, with two thirds of these losses occurring at the NRC (Table 2).^24^ More recent data from this site indicate a stable federal workforce since 2016. The NRC reductions were attributed, in part, to staff downsizing associated with the cancellation of new reactor projects and the closing of 13 power plants since 2013. Of note, some federal departments, such as DOE, have used outsourcing to augment the capacity of their professional HP staff.

**TABLE 2:** Distribution of health physicists by employment sector^12^, ^a^

Sector	Number of HPs	%
Government	447	20.9
Industrial	283	13.2
Medical	271	12.7
Military	50	2.3
National laboratory	210	9.8
Private practice	237	11.1
University	312	14.6
Other	328	15.3

^a^ Based on a survey of HPS members (2138 respondents).

State radiation control programs, which are monitored and periodically reviewed by the NRC, appear to currently have an adequate health physics workforce. These programs employ ∼1000 full‐time equivalent employees, although the actual number of employees is uncertain as personnel are assigned across multiple public health duties simultaneously.^7^ In addition, these programs are challenged by attrition due to staff leaving state service for higher paying federal or private sector positions, which in turn necessitates the training of replacement staff to qualify for licensing, inspection or compliance work. The NRC provides the training and travel funds needed to meet these radiation control program requirements. A representative of the Conference of Radiation Control Program Directors echoed the concern of many at the 2013 NCRP WARP Workshop regarding the impact of large‐scale retirements without an adequate pool of replacements and concluded that it may be necessary in the future to train general science graduates to perform radiation protection duties.^25^

A workshop explored areas of health physics expertise that will be required to fulfill research needs.^26^ This focused on research needs and did not cover workforce issues. It was observed that, across many academic programs, alternative, non‐radiological technologies have significantly reduced the use of radioactive materials in biomedical research, decreasing the need for health physics staff.

Recent unpublished data from Little and Johnson compiled job announcements from Colorado State University alumni, as well as from the “Indeed” website from June 2020 to February 2021 (Little and Johnson, email communication, 5 May, 2022) (Table 3). A period of 8 months of data collection yielded 643 unique job announcements for HPs. Of these, only 125 were specifically denoted as technician level positions. A total of 194 were specifically announced as jobs for “HP,” with 39 others titled “RSO.” Other job titles varied from “Environmental Scientist” (typically for state level health physics positions, *n* = 27 announcements) to “Physicist” (*n* = 20). Job descriptions were carefully examined to ensure that nuclear engineering and medical physics positions were not misclassified as health physics. The preliminary analysis indicated an average of 80 unique postings per month, with ∼16 for technicians and 64 for professional HPs. Analysis of previous years is ongoing; however, based on the quantity of data, similar numbers are expected for 2019 and 2018. These data suggest a robust demand for HPs, and anecdotal information indicates that competition for the limited pool of graduates is high.

**TABLE 3:** Count of radiation protection job postings, June 2020 to February

Military	37
Federal govt	34
State govt	58
National lab	58
Medical	22
Industry	176
University	22
Other	236
**Total**	643

## 2.6 SUMMARY AND RECOMMENDATIONS

We conclude that the demand for HPs is sector‐ and specialty‐specific and subject to varied and multiple factors and external influences. One example of this is driven by the shuttering of nuclear power plants; as they cease generating power, new and different works are necessary for the decontamination and decommissioning phase, requiring a reallocation of HP resources. This makes the assessment of workforce needs very challenging. Although a review of the published literature revealed a lack of evidence in support of earlier predicted shortages,^3^ new data suggest that there may in fact be current pent‐up demand for radiation protection specialists that had not previously been recognized. It is quite possible that major shortages were averted by a drop in demand in certain sectors (e.g., nuclear power) and the delayed retirements of a significant segment of the workforce. However, the data available on the demographics of the professional health physics workforce indicate that the profession will experience an unprecedented wave of attrition as baby boomers leave the workforce. The future impacts of the COVID‐19 pandemic are unknown. The demand for recent graduates demonstrates the need to provide continued support for health physics training programs. In addition, although certain radiation protection operations may be performed by generalists under ideal conditions, it is clear that professional HPs are needed for accidents or other emergencies involving radiological or nuclear activities. Furthermore, it must be acknowledged that institutional knowledge, once lost, is difficult to regain, and efforts should be made to retain and transfer this knowledge to the next generation of professional HPs.

More and better quality data on the health physics workforce are needed. In particular, more frequent and focused studies are needed to adequately characterize the supply and demand for health physics professionals to ensure that the nation's future needs will be met. Although data are collected annually on health physics education and employment, longer range forecasting of changes in supply (e.g., due to attrition) and demand (e.g., due to changes in utilization of radiation sources) are notoriously difficult because of the large impact of unpredictable factors, such as economic conditions. Hence, annually updated, short‐term forecasts are indicated to inform decision‐making regarding the workforce.

The recommendations later represent consensus expert opinions on actions needed to ensure that the health physics profession will be able to meet the nation's future needs. The Committee intentionally declined to recommend detailed methods, timelines, responsibilities of individual organizations, and funding sources. These complex subjects are outside the scope of this Review and, indeed, the Committee was prohibited from activities that could be construed as advocacy.

**The authors recommend the following actions to ensure the future adequacy of the nation's professional health physics workforce**:

1. Conduct comprehensive studies to accurately assess the current health physics workforce.
  - Increase the scope, specificity, and granularity of workforce surveillance.
  - Study each employment sector and subspecialty in detail to obtain an accurate count of current HP positions.
  - Based on consistent and adequate survey from annual surveys, longitudinal analyses should be performed to better understand long‐term trends.
  - Coordinate and collaborate with survey efforts of other radiation professions in order to enhance the direct comparability of survey data, for example, especially with the closely related profession of medical physics.
2. Assess the supply of health physics professionals.
  - Determine current gaps in employment across all sectors and subspecialties.
  - Conduct detailed assessments of health physics education and training programs to project future supply.
  - Increase outreach to young students to encourage entry into the health physics field.
3. Conduct detailed analyses to support forecasting of employment needs by sector.
  - Increase the frequency and accuracy of workforce projections.
  - Compile and categorize vacancy announcements.
  - Projections should attempt to improve prediction of future changes in demand, which appears to have played a role in some projections that were performed previously.
4. Stabilize and support education and training programs for HPs.
  - Establish consistent and rigorous standards for minimum education, training, qualifications, and experience for health physics employment.
  - Stabilize or increase funding and other support for education and training programs.
  - Stabilize declining enrollments in and graduations from health physics degree programs.
    i. Provide support for alternate training programs and pathways.
    ii. Increase funding for health physics workforce development activities, including support for higher education programs, fellowships and scholarships for students (undergraduate and graduate level), research, curriculum development, and faculty development (hiring).
    iii. Foster increased utilization of university–employer partnerships, for example, co‐op and internship and externship opportunities, to increase alignment of university curricula with the employers’ current and future needs.
  - Prioritize succession planning to account for impending retirements.
  - Consider a centralized organization to coordinate force management and career development.
5. Maintain a cadre of highly trained and qualified experts in radiological/nuclear emergency preparedness and response.

## AUTHOR CONTRIBUTION

All the authors listed have contributed directly to the intellectual content of the manuscript.

## CONFLICT OF INTEREST

No conflict of interest.
